# The role of the hyoid bone in mandibular advancement: insights from functional appliance therapy in patients with skeletal class II – a prospective cohort study

**DOI:** 10.1186/s13005-025-00552-3

**Published:** 2025-11-15

**Authors:** Janine Sambale, Vivian La Garde, Ulrich Koehler, Heike Maria Korbmacher-Steiner

**Affiliations:** 1 Department of Orthodontics, Clinic of Dentistry, Marburg University, Georg-Voigt-Str. 3, Marburg, 35039 Germany; 2 Department of Pneumology, Interdisciplinary Center of Sleep Medicine, Marburg University, Marburg, 35039 Germany

**Keywords:** Class II, Hyoid bone position, Functional appliance, Mandibular advancement, Lateral cephalography, Diagnosis

## Abstract

**Study objectives:**

Functional orthopedics induces mandibular advancement caused by neuromuscular adaptations. These adaptations can affect the position of the hyoid bone and may contribute to the amount of skeletal outcomes. The skeletal response is triggered by the quality and quantity of growth as well as muscular reactions. The aim of this clinical trial was to evaluate the hyoid bone position before and after functional orthodontic treatment.

**Methods:**

This prospective cohort study included 31 patients (mean age: 12.3 ± 0.9 years) with skeletal class II meeting the following inclusion criteria: ANB > 4°, > ½ Class II molar relationship, overjet > 6 mm, neutral/horizontal growth pattern, and CVMS II–III. All patients were treated with the Sander Bite Jumping Appliance (BJA) with wear time monitored through microsensors. Lateral cephalograms were taken at baseline (t0) and after 1 year (t1). Linear and angular hyoid measurements were analyzed. Statistical analysis was performed using IBM SPSS (v29.0.2.0), employing paired t-tests and an exploratory post hoc grouping based on total mandibular length changes (Δco-pg), with significance set at *p* < 0.05.

**Results:**

BJA therapy resulted in a significant more superior and anterior position of the hyoid in all patients. A greater increase in Δco-pg at t1 was associated with a more pronounced hyoid shift, while patients with a less Δco-pg showed a more inferior hyoid position at t0 and a greater tendency towards a vertical growth pattern.

**Conclusion:**

A more inferior initial hyoid position was associated with limited mandibular advancement, suggesting a potential anatomical link to airway physiology, warranting further investigation into its implications for OSA risk.

**Clinical trial registration:**

German Clinical Trials Register (DRKS); URL: http://www.germanctr.de; Identifier: DRKS00021090; registration date: 12.03.2020.

## Introduction

The hyoid bone is a freely moveable U-shaped structure located in the anterior region of the neck between the mandible and the thyroid cartilage. Unlike most bones, it is not directly connected with any other bones [1]. Instead, it is suspended by muscles, allowing complex movements during breathing, swallowing and changes in gravitational orientation. Its anterior and lateral aspects contribute to the rigidity of the hypopharynx, thus its position and its movements changes the size and shape of the upper airway. Hyoid muscles seem to have a stabilizing and dilating effect on the pharynx with several studies showing an increased ability of the upper airway to resist pharyngeal collapse when the hyoid bone was tracted anteriorly [2–4].

The position of the hyoid bone is determined by the muscle force vectors between supra- and infrahyoid muscles and is critical in the pathogenesis of obstructive sleep apnea (OSA) as its caudal displacement can exacerbate airway obstruction and increase the risk of apneic events [5–7].

There is increasing recognition of the orthodontist’s role in screening and managing craniofacial risk factors for OSA [8]. OSA is a common sleep-related breathing disorder characterized by recurrent episodes of partial or complete obstruction of the pharyngeal airway during sleep, typically caused by pharyngeal narrowing and collapse [9]. The pathophysiology of OSA is multifactorial, involving craniofacial and neuromuscular components and other related factors [10]. Craniofacial characteristics such as a skeletal class II with mandibular retrognathia, a dolichocephalic facial pattern, and an inferiorly positioned hyoid bone are recognized as substantial risk factors [11–13]. If uncorrected during growth, these structural risk factors may persist into adulthood [14, 15].

Functional orthopedics, along with interventions such as myofunctional therapy can contribute to both prevention and treatment in children [8]. Functional orthodontic appliances that stimulate mandibular advancement through forward positioning of the mandible represents an important preventive and therapeutic approach, particularly in growing patients with retrognathic mandibles. By repositioning the mandible anteriorly, these appliances stimulate adaptive changes in the orofacial musculature and temporomandibular complex. Anterior displacement alters neuromuscular activation patterns, particularly in protrusive muscles such as the lateral pterygoid, geniohyoid, and mylohyoid, and can lead to skeletal adaptations as well as soft tissue modifications, including those affecting the hyoid bone [16, 17]. When applied during pubertal growth, such therapy has been shown to influence the position of the hyoid bone, which could contribute to enhanced airway stability [1, 18, 19]. Early orthodontic intervention targeting these craniofacial abnormalities can prevent the development of long-term respiratory dysfunction [8].

The treatment success with functional orthopedics is strongly influenced by factors such as growth pattern, lip posture, treatment timing, and adherence [20]. A horizontal or neutral growth pattern, characterized by counterclockwise (ccw) or neutral mandibular rotation, is associated with more favorable skeletal outcomes, whereas a vertical growth pattern with clockwise (cw) rotation often leads to further cw rotation during treatment [21, 22].

In contrast, the potential role of the hyoid bone as a predictive factor in mandibular advancement with functional appliances has received little attention.

Therefore, the present study aimed to evaluate changes in the position of the hyoid bone in patients with skeletal class II following functional orthodontic treatment and to assess the relationship between the hyoid bone position and changes in mandibular length. We hypothesized that there is an association between the skeletal mandibular outcome and the position of the hyoid bone.

## Subjects and methods

### Study design

This prospective cohort study analyzed the position of the hyoid bone before and after orthodontic functional appliance therapy in relation to the changes of mandibular length after functional treatment.

Ethical approval was obtained from the Ethics Committee of University Marburg (approval number 145/19) in accordance with the Declaration of Helsinski and was registered in the German Clinical Trials Register (DRKS00021090, date of registry: 12 March 2020).

A sample size calculation was performed using G*Power (Version 3.1). Based on an expected mean change of the hyoid of 2 mm with a standard deviation of 3 mm, a dependent samples t-test was used as the statistical model.

To achieve a statistical power of 95% and a significance level of α = 0.05, the required sample size was calculated to be 26 patients. Considering an estimated dropout rate of 20%, it was determined that at least 31 patients should be included. The assumption of a clinically relevant change of 2 mm is supported by the findings of Hourfar et al. [19], who investigated hyoid position changes following treatment with two different fixed functional orthodontic appliances. Their results demonstrated that both appliances led to a mean anterior–posterior displacement of the hyoid by approximately 2 mm relative to the anterior borders of the cervical vertebrae C2, C3, and C4. These findings suggest that a 2-mm change represents a realistic and clinically meaningful treatment effect.

In total, 42 patients were recruited at the Department of Orthodontics, Institute of Dentistry, University of Marburg, between 2020 and 2023.

**Fig. 1:**
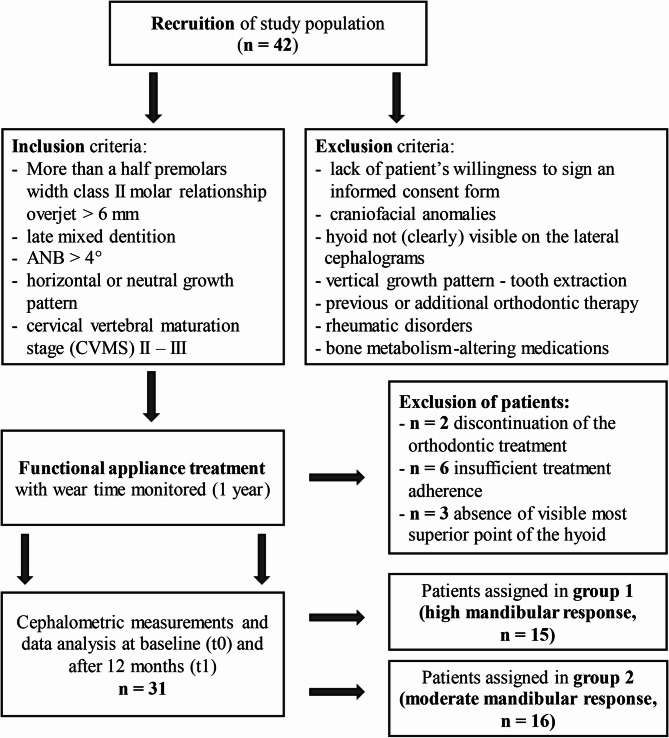
Flowchart of study population

According to a first analysis the inclusion criteria were as follows: more than a half premolars width class II molar relationship (i.e., >3.5 mm), overjet >6 mm, late mixed dentition, ANB >4°, horizontal or neutral growth pattern (sum of Björk polygon angles ≤ 401°), and cervical vertebral maturation stage (CVMS) II – III [20, 23]. The exclusion criteria were lack of patient’s willingness to sign an informed consent form, craniofacial anomalies, os hyoideum with its most superior-anterior point (H) not (clearly) visible on the lateral cephalograms, vertical growth pattern (sum of Björk polygon angles: >401°), tooth extraction, previous or additional orthodontic therapy, rheumatic disorders, and bone metabolism-altering medications. A study flowchart is presented in Fig. 1.

Pre-treatment anamnesis revealed no clinical signs of sleep-disordered breathing, such as habitual loud snoring or excessive daytime sleepiness. Consequently, all patients were considered sleep-healthy, and no indication for polygraphic or polysomnographic assessment was present prior to or following functional appliance therapy.

Two orthodontists (JS and HMKS) participated in the recruitment process and the treatment. Before the beginning of the study, they were calibrated to ensure consistency in both methodological and clinical procedures.

## Participants

### Functional appliance therapy

The skeletal Class II malocclusion was corrected using the Sander bite-jumping appliance (BJA). The treatment protocol involved the use of the BJA and was constructed according to the method described by Gazzani et al. [24] For patients with an initial transverse discrepancy, the upper jaw’s expansion screw was activated at a rate of one turn per week (0.25 mm). In cases of lingual tipping of the lower molars, the lower jaw’s expansion screw was adjusted more gradually, with one turn per month (0.25 mm). Adjustments to the lower expansion screw were specifically aimed at leveling the Wilson curve. The desired therapeutic mandibular position was recorded three-dimensionally using a wax construction bite with a single-step mandibular advancement. In the sagittal dimension, the mandible was advanced to achieve a super-Class I molar relationship. Transverse discrepancies, including gnathic midline deviations, were corrected in the transverse plane, while a 2-mm anterior vertical opening was established in the vertical plane. Activation of the transversal screws affected only incisor position if it was necessary, otherwise the labial bow was deactivated during the expansion period. The bite registration, which ensured a super-Class I molar relationship, determined whether activation or deactivation of the upper labial bow was required and guided further adjustments to achieve a physiological overjet of 2 mm by reclining the upper incisors. To minimize undesired dental effects such as proclination of the lower incisors, lingual reduction of the lower plate was performed in all patients, and the labial bow was activated. Patients were instructed to wear the appliance for more than 12 h daily, with adherence objectively monitored through a temperature-sensitive microsensor (TheraMon^®^, MC Technology GmbH, Austria) embedded in the upper plate. Follow-up appointments were scheduled every six weeks to record wear time using a TheraMon^®^ pen. Adherence data were shared with patients during visits to motivate them to maintain adequate wear times. Patients with an average daily wear time below 10 h were excluded from the study. At treatment completion after about one year functional appliance treatment (t1), either a Class I or super-Class I molar relationship was achieved, ensuring that mandibular retraction was no longer possible.

### Cephalometric analysis

**Fig. 2:**
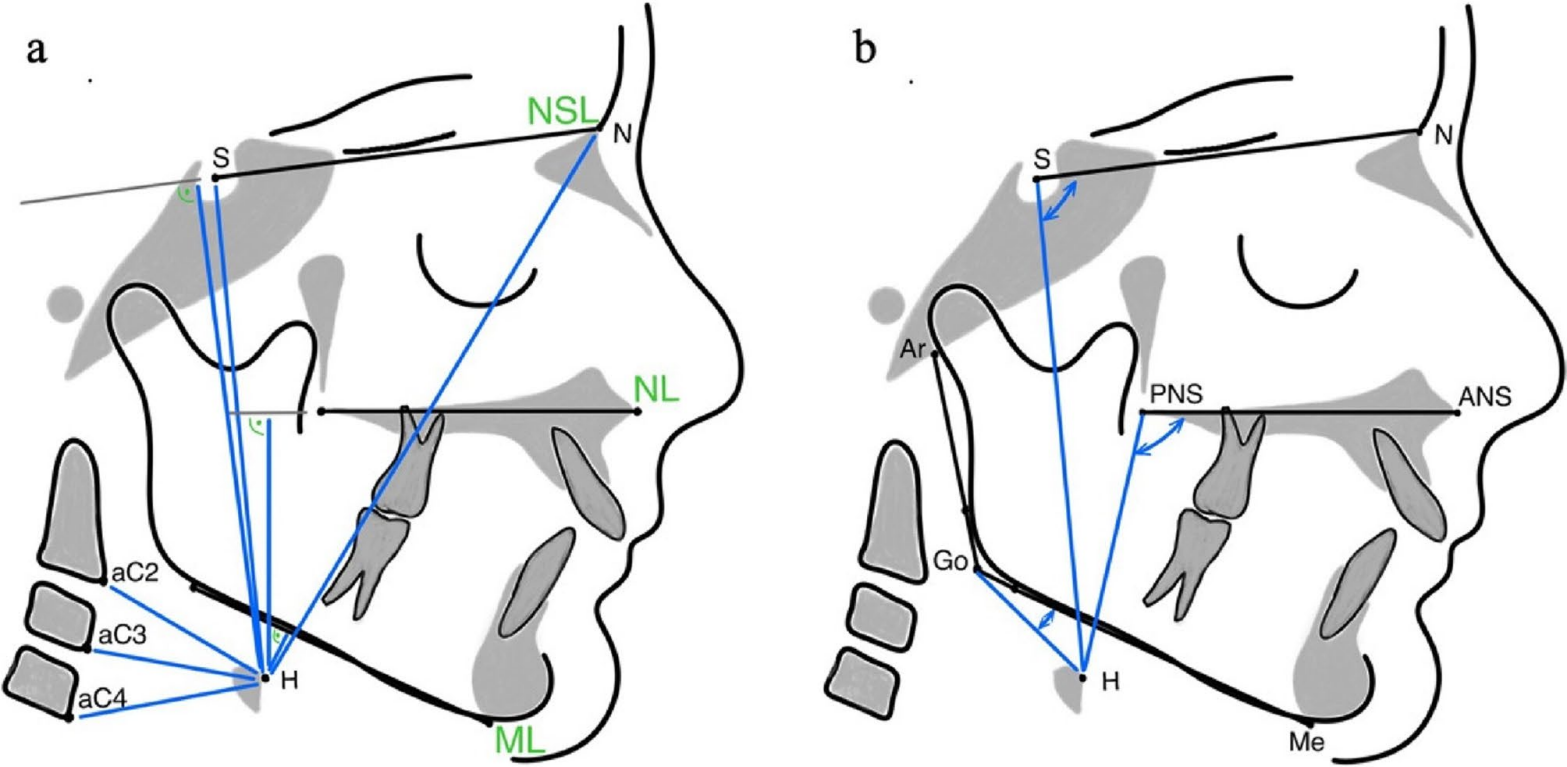
Hyoid bone (H). **A**: Linear measurements from H (H-aC2, H-aC3, H-aC4, H-S, H-N, H-NL, H-NSL). **B**: Angular measurements from H (N-S-H, ANS-PNS-H and Me-Go-H)

**Fig. 3:**
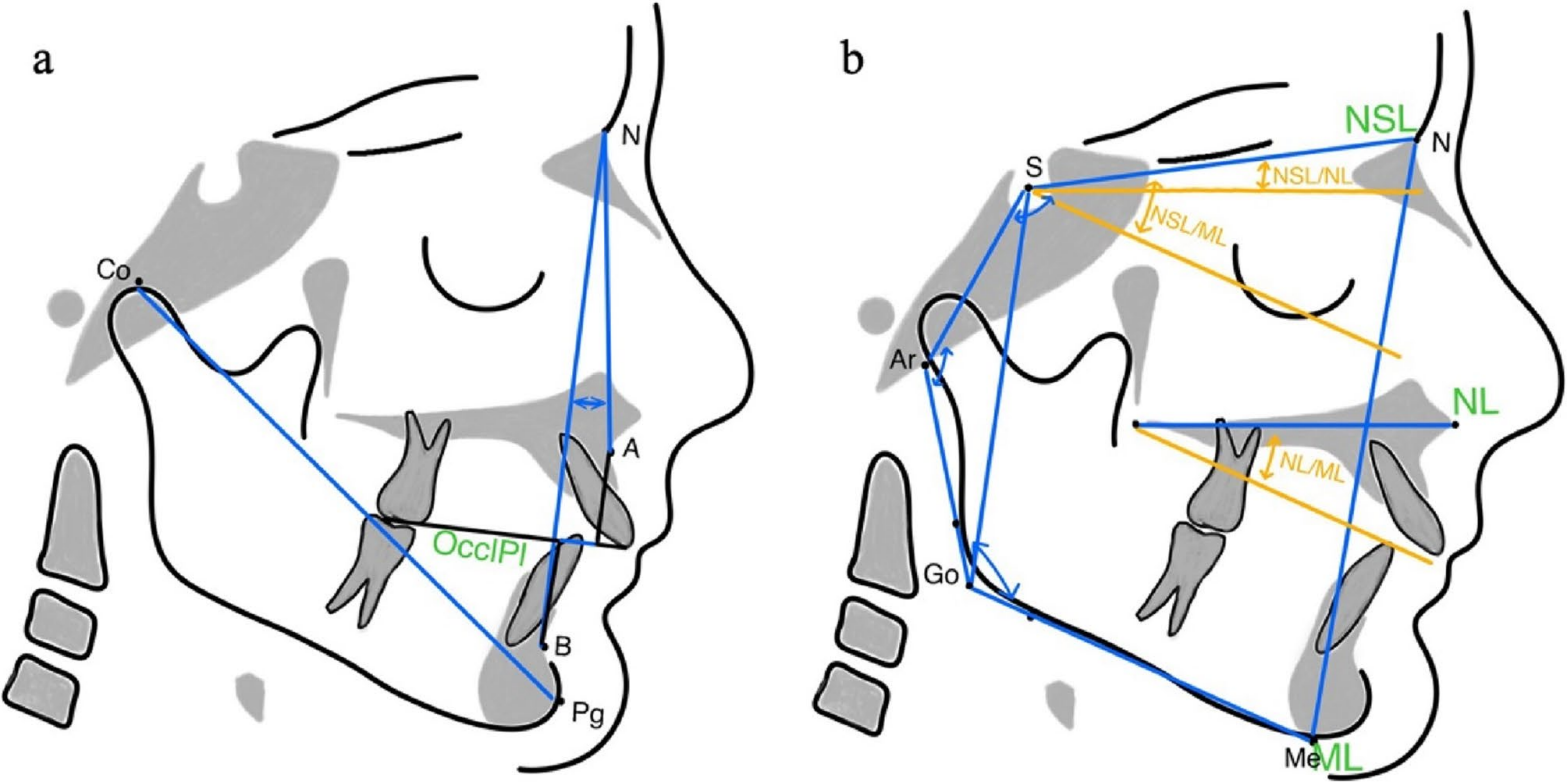
Craniofacial characteristics. **A**: Linear measurement of total mandibular length (co-pg) and linear (Wits appraisal) and angular (ANB) sagittal measurements of intermaxillary relationship. **B**: Linear (Jarabak Ratio) and angular (Sum of Björk polygon angles, NSL-ML, NL-ML, NSL-ML) vertical characteristics

Exposure values for the lateral cephalograms (PLANMECA, ProMax) were set to 66–68 kV and 5 mA depending on the patient’s size. Patients were secured with a nasal rest to Nasion (N) and head inclination was adjusted according to the Frankfurt horizontal plane. Lateral cephalograms were taken in centric occlusion with lips in the resting position at t0 and t1. Both lateral cephalograms were conducted as part of the routine treatment of the functional orthopedic treatment. Before the examination the cephalograms were standardized using a magnification factor and were blinded to the patients’ name and allocation. Tracing and measurements were analyzed manually. To ensure quality of data assessment the examiner (VD) was extensively trained by one author (JS). After analyzing all the 62 cephalograms VD reanalyzed 20 randomized cephalograms of 10 patients (t0 and t1) three weeks later according to the intraexaminer consistency and reproducibility. According to the interexaminer agreement JS analyzed independently 20 randomized cephalograms of 10 patients (t0 and t1). The definitions of all cephalometric measurements are shown in Table 1. Linear and angular measurements from the hyoid bone are presented in Fig. 2. The linear and angular measurements of the sagittal and vertical craniofacial characteristics are represented in Fig. 3.

**Table 1:** Definition of cephalometric measurements

Measurement	I. Hyoid bone
Linear (mm)	
H-aC2	Distance between the most anterior and most superior point of the hyoid bone (= H) and the anterior border of the second cervical vertebra (= aC2)
H-aC3	Distance between the most anterior and most superior point of the hyoid bone (= H) and the anterior border of the third cervical vertebra (= aC3)
H-aC4	Distance between the most anterior and most superior point of the hyoid bone (= H) and the anterior border of the fourth cervical vertebra (= aC4)
H-N	Distance between the most anterior and most superior point of the hyoid bone (= H) and the intersection of the nasofrontal suture in the mid-sagittal plane (= N)
H-S	Distance between the most anterior and most superior point of the hyoid bone (= H) and the centre of the pituitary fossa of sphenoid bone (= S)
H-NL	Perpendicular distance from the most anterior and superior point of the hyoid bone (= H) to the maxillary plane (= NL, defined as the line connecting the anterior nasal spine (= ANS) to the posterior nasal spine (= PNS)
H-NSL	Perpendicular distance from the most anterior and superior point of the hyoid bone (= H) to the cranial base (= NSL, defined as the line passing through N and S)
H-ML	Perpendicular distance from the most anterior and superior point of the hyoid bone (= H) to the mandibular plane (= ML, defined as a line passing through Go and Me)
Angular (°)	
Me-Go-H	Angle between the most inferior point of the symphysis (= Me), the most posterior-inferior point at the angle of the mandible constructed as the junction of the posterior and inferior borders of the mandibular ramus (= Go) and most superior point of the hyoid bone (= H)
ANS-PNS-H	Angle between the tip of the anterior nasal spine (= ANS), the tip of the posterior nasal spine (= PNS) and the most anterior and most superior point of the hyoid bone (H)
N-S-H	Angle between the intersection of the nasofrontal suture in the mid-sagittal plane (= N), the centre of the pituitary fossa of sphenoid bone (= S) and the most anterior and most superior point of the hyoid bone (= H)

Measurement	II. Craniofacial characteristics
Linear (mm)	
Co-pg	Total mandibular length of the distance between co (= condylar head as the most superior point of the mandibular condyle) and pg (= pogonion as the most anterior point on the bony contour of the chin)
Wits appraisal	Distance between perpendicular projections of points A (= the most concave point on the anterior surface of the maxilla) and B (= the most concave point on the anterior contour of the mandible) and in the occlusal plane (OcclPl)
Angular (°)	
ANB	Anteroposterior relationship between the maxilla and mandible calculated as the difference between SNA (= sella-nasion to A-point) and SNB (sella-nasion to B-point)
Sum of Björk polygon angles	The sum of the saddle angle (N-S-Ar), the articular angle (S-Ar-Go), and the gonial angle (Ar-Go-Me)
NSL/ML	Inclination of the mandibular base (Mandibular Line, ML) relative to the cranial base (Nasion-Sella Line, NSL)
NSL/NL	Inclination of the maxillary base (Nasal Line, NL) relative to the cranial base (Nasion-Sella Line, NSL)
NL/ML	Vertical relationship between the nasal line (NL) and the mandibular line (ML)
Jarabak Ratio	Ratio between posterior facial height (measured from sella to gonion) and anterior facial height (measured from nasion to menton)

## Statistical analyses

Data analysis was performed using IBM SPSS (V. 29.0.2.0). The Shapiro-Wilk test was performed on all variables to test for normality of distribution. Data were analyzed with proportions (number of patients), arithmetic means, standard deviations and median values. Dependent samples t-tests were used for the total sample size to analyze changes between t0 and t1. The median Δco-pg was chosen as a cut-off to allow for an exploratory, post-hoc data-driven stratification of patients into equally sized subgroups, ensuring balanced statistical power to evaluate patients with higher vs. lower mandibular length response. Group 1 was defined as the high mandibular response group, while group 2 was defined as the moderate mandibular response group. The independent samples t-test were used for group comparisons. A *p* value of ≤ 0.05 was considered statistically significant. Intaclass correlation coefficient (ICC) estimates and their 95% confident intervals (CI) were calculated for intrarater and interrater reliability.

## Results

Of the 42 patients initially included in the study, 11 were excluded for the following reasons: discontinuation of the orthodontic treatment in 2 patients, insufficient treatment adherence in 6 patients (mean wearing time < 10 h per day), and the absence of visible most superior point of the hyoid bone in 3 patients.

Intra- and interexaminer reliability values showed excellent agreements (ICC_intra_ = 0.96; [CI: 0.91; 0.98]; ICC_inter_ = 0.96; [CI: 0.91; 0.99]) [25].

A total of 31 patients (21 female, 10 male) were included in the statistical analysis. The mean chronological age was 12.3 ± 0.9 years, with a mean treatment duration of 1.1 ± 0.1 years and a mean wearing time of 10.9 ± 0.8 h per day.

The significant increase in co-pg (+ 5.23 ± 3.00 mm, *p* < 0.001), as well as the significant reduction in ANB (−1.63 ± 0.63 °, *p* < 0.001) and Wits appraisal (− 2.77 ± 1.02 mm, *p* < 0.001) reflects both the effect of BJA treatment and the contribution of mandibular growth during the observation period compared with an untreated control group published by Baccetti et al. [26] (Table 2).

Pretreatment hyoid position data (t0) and treatment-related changes in hyoid position (Δ t1 - t0) are presented in Table 3. BJA treatment resulted in significant linear increases of H-aC2 (*p* = 0.018), H-aC3 (*p* < 0.001), H-aC4 (*p* < 0.001), H-NSL (*p* < 0.001), H-NL (*p* = 0.012), H-S (*p* = 0.001) and linear decreases in H-ML (*p* = 0.003). Additionally, significant angular changes were observed in Me-Go-H (*p* = 0.017).

**Table 2:** Pre- (t0) and posttreatment (t1) data of the sagittal and vertical skeletal craniofacial growth pattern

Craniofacial growth pattern	Total sample size			
	t0	t1	Δ t1 - t0	*P* value
	Mean (SD)	Mean (SD)	Mean (SD)	
Sagittal plane				
Co-pg (mm)	99.57 (7.61)	104.94 (8.10)	+ 5.23 (3.00)	< 0.001***
ANB (°)	5.40 (1.18)	3.74 (1.16)	− 1.63 (0.63)	< 0.001***
Wits (mm)	3.24 (1.53)	0.47 (1.22)	− 2.77 (1.02)	< 0.001***
Vertical plane				
Sum of Björk polygon angles (°)	390.41 (4.15)	391.71 (4.48)	+ 1.33 (2.18)	0.003**
NSL/ML (°)	30.42 (4.16)	31.69 (4.47)	+ 1.27 (2.25)	0.095^n.s^.
NSL/NL (°)	7.21 (3.43)	7.87 (4.15)	+ 0.67 (2.20)	0.101^n.s^.
ML/NL (°)	23.20 (4.30)	23.81 (4.42)	+ 0.61 (1.97)	0.004**
Jarabak Ratio (mm)	67.46 (3.69)	67.58 (4.07)	+ 0.12 (2.13)	0.757^n.s^.

*Abbr*.: *SD* standard deviation, *n.s.* not significant

** *p* < 0.01

*** *p* < 0.001

**Table 3:** Pre- (t0) and posttreatment (t1) hyoid position and it’s treatment related changes (Δt1 - t0) of the total sample size

Measurement	Total sample size			
	t0	t1	Δ t1 - t0	*P* value
	Mean (SD)	Mean (SD)	Mean (SD)	
Linear (mm)				
H-aC2	32.40 (4.74)	33.54 (4.34)	+ 1.14 (2.52)	0.018*
H-aC3	28.39 (3.49)	30.83 (3.49)	+ 2.44 (2.36)	< 0.001***
H-aC4	32.64 (3.69)	35.86 (4.41)	+ 3.22 (3.67)	< 0.001***
H-ML	12.46 (4.89)	10.77 (4.56)	− 1.68 (2.89)	0.003**
H-NSL	91.60 (7.01)	94.18 (7.03)	+ 2.59 (3.52)	< 0.001***
H-NL	51.92 (4.97)	53.37 (5.35)	+ 1.45 (3.03)	0.012*
H-N	115.07 (6.22)	117.21 (7.11)	+ 2.14 (7.54)	0.125^n.s^.
H-S	92.22 (6.84)	95.28 (8.29)	+ 3.06 (3.88)	< 0.001***
Angular (°)				
Me-Go-H	25.82 (9.03)	23.33 (8.71)	− 2.49 (5.50)	0.017*
ANS-PNS-H	99.80 (5.97)	98.85 (6.99)	− 0.13 (3.99)	0.191^n.s^.
N-S-H	91.91 (5.57)	91.78 (5.30)	− 0.13 (3.48)	0.838^n.s^.

*Abbr*.: *SD* standard deviation, *n.s.* not significant

*p < 0.05

**p < 0.01

***p < 0.001

## Group comparison

The median total mandibular length response (Δ co-pg) was + 4.84 mm. Based on the Δ co-pg values, the patients were divided into two groups: Group 1 (Δ co-pg ≥ + 4.84 mm; *n* = 15), referred to high mandibular response group, and group 2 (Δ co-pg < + 4.84 mm; *n* = 16), referred to moderate mandibular response group according to Baccetti et al. [26].

At t0, there were no significant differences in terms of sex (*p* = 0.314), skeletal (*p* = 0.463), chronological age (*p* = 0.149), and wearing time (*p* = 0.433) between both groups.

Pretreatment (t0) hyoid position measurements and treatment related changes are summarized in Table 4. At t0 group 2 exhibited higher vertical values for H-ML, H-NSL, H-NL, H-N, and H-S, as well as higher sagittal values for H-aC2, H-aC3 and H-aC4 compared to group 1.

During treatment, group 1 demonstrated a statistically significant increase in sagittal hyoid position parameters (H-aC2, H-aC3, and H-aC4), with greater treatment-related changes compared to group 2. In contrast, angular hyoid measurements in group 2 revealed non-significant changes, with smaller decreases observed compared to group 1.

**Table 4:** Intergroup comparison of pre- (t0) and posttreatment (t1) hyoid position and it’s treatment related changes (Δt1 - t0)

Measurement	High mandibular response (group 1)				Moderate mandibular response (group 2)				Group comparison
	t0	t1	Δ t1 - t0	*P* value^a^	t0	t1	Δ t1 - t0	*P* value^a^	*P* value^b^
	Mean (SD)	Mean (SD)	Mean (SD)		Mean (SD)	Mean (SD)	Mean (SD)		
Linear (mm)									
H-aC2	32.06 (5.45)	33.27 (4.61)	+ 1.21 (2.98)	0.010*	32.72 (4.13)	33.79 (4.21)	+ 1.08 (2.11)	0.060^n.s^.	0.196
H-aC3	27.80 (3.26)	30.60 (3.36)	+ 2.80 (2.64)	0.001**	28.93 (3.72)	31.04 (3.71)	+ 2.11 (2.09)	0.001**	0.371
H-aC4	31.41 (2.98)	35.30 (2.97)	+ 3.89 (3.98)	0.002**	33.80 (4.00)	36.38 (5.49)	+ 2.58 (3.37)	0.008**	0.490
H-ML	11.95 (4.99)	10.41 (4.33)	− 1.55 (2.60)	0.026*	12.93 (4.91)	11.12 (4.89)	− 1.81 (3.22)	0.040*	0.485
H-NSL	90.68 (7.37)	93.49 (7.69)	+ 2.81 (3.55)	0.006**	92.46 (6.78)	94.84 (6.54)	+ 2.38 (3.59)	0.018*	0.295
H-NL	51.29 (4.31)	52.89 (4.92)	+ 1.61 (3.22)	0.092^n.s^.	52.51 (5.59)	53.81 (5.85)	+ 1.30 (2.94)	0.097^n.s^.	0.396
H-N	113.95 (5.55)	116.13 (6.78)	+ 2.19 (5.26)	0.103^n.s^.	116.13 (6.80)	118.21 (7.48)	+ 2.09 (9.37)	0.387^n.s^.	0.322
H-S	91.43 (7.16)	93.94 (7.52)	+ 2.51 (4.07)	0.021*	92.96 (6.67)	96.53 (9.01)	+ 3.58 (3.76)	0.002**	0.292
Angular (°)									
Me-Go-H	25.32 (9.73)	22.02 (8.00)	−3.30 (5.92)	0.035*	26.29 (8.62)	24.56 (9.42)	−1.73 (5.15)	0.199^n.s^.	0.054
ANS-PNS-H	100.98 (5.42)	99.54 (4.63)	− 1.44 (4.50)	0.046*	98.70 (6.42)	98.19 (8.76)	− 0.51 (3.53)	0.574^n.s^.	0.073
NSH	90.79 (5.10)	90.39 (5.15)	− 0.40 (2.10)	0.412^n.s^.	92.96 (5.94)	93.08 (5.26)	+ 0.13 (4.46)	0.912^n.s^.	0.120

*Abbr*.: *SD* standard deviation, *n.s.* not significant

^a^dependent samples t-test

^b^independent samples t-test

*p < 0.05

**p < 0.01

The intergroup comparison of pre- (t0) and posttreatment (t1) vertical skeletal pattern, as well as the treatment related changes (Δ t1 - t0) are presented in Table 5.

At t0 group 2 exhibited higher values of Björk polygon angles, NSL/ML, NSL/NL and NL/ML, along with a lower Jarabak Ratio compared to group 1. At t1, group 2 showed a significant increase in Björk polygon angles and NSL/ML.

**Table 5:** Intergroup comparison of pre- (t0) and posttreatment (t1) vertical skeletal pattern and their treatment related changes (Δ t1 - t0)

	High mandibular response (group 1)				Moderate mandibular response (group 2)				Group comparison
Vertical skeletal pattern	t0	t1	Δ t1 - t0	*P* value^a^	t0	t1	Δ t1 - t0	*P* value^a^	*P* value^b^
	Mean (SD)	Mean (SD)	Mean (SD)		Mean (SD)	Mean (SD)	Mean (SD)		
Angular (°)									
Sum of Björk polygon angles	389.81 (4.30)	390.87 (4.06)	+ 1.05 (2.20)	0.072^n.s^.	390.98 (4.06)	392.49 (4.40)	+ 1.56 (2.20)	0.018*	0.265
NSL/ML	29.81 (4.30)	30.82 (4.52)	+ 1.01 (2.24)	0.088^n.s^.	30.98 (4.07)	32.50 (4.42)	+ 1.52 (2.31)	0.019*	0.535
NSL/NL	6.61 (4.06)	7.15 (5.03)	+ 0.54 (1.93)	0.427^n.s^.	7.77 (2.72)	8.56 (3.13)	+ 0.79 (2.48)	0.223^n.s^.	0.379
NL/ML	23.20 (4.54)	23.65 (4.42)	+ 0.45 (1.86)	0.235^n.s^.	23.45 (4.30)	24.21 (4.57)	+ 0.76 (2.12)	0.174^n.s^.	0.337
Linear (mm)									
Jarabak Ratio	67.72 (4.14)	68.32 (4.71)	+ 0.60 (2.55)	0.429^n.s^.	67.22 (3.34)	66.89 (3.37)	− 0.33 (1.61)	0.423^n.s^.	0.115

*Abbr*.: *SD* standard deviation, *n.s.* not significant

^a^dependent samples t-test

^b^independent samples t-test

*p < 0.05

## Discussion

This prospective cohort study examined the position of the hyoid bone in patients with skeletal class II malocclusion and mandibular retrognathism before and after functional orthodontic treatment. To the best of our knowledge, this study is the first to quantitatively assess the relationship between the initial position of the hyoid bone and treatment-induced changes in total mandibular length.

In this study, we observed a cranial and anterior displacement of the hyoid bone following functional appliance therapy. This can be attributed to the hyoid bone’s anatomical connections with the mandible via the geniohyoid, mylohyoid and anterior belly of the digastric muscles. Functional appliances increase the activity of the suprahyoid muscles, leading to neuromuscular reorganization and stimulating the activity of the mandibular protractor muscles while inhibiting the retractors simultaneously [27]. Such changes have been documented in electromyographic studies demonstrating enhanced activity of the genioglossus and mylohyoid muscles during functional appliance therapy [17, 21].

While several previous studies have reported an inferior displacement of the hyoid following functional orthopedic therapy, these discrepancies can largely be attributed to variations in cephalometric reference points. In this study, we used the distance from the hyoid to the mandibular plane (H-ML) as our primary measurement parameter, as it is less influenced by vertical growth and more accurately reflects biomechanical changes induced by functional appliances.

For instance, Hourfar et al. [19] reported that, after functional orthodontic treatment, the hyoid assumed a more inferior and ventral position. However, in contrast to our study cohort, which was treated with the same removable functional appliance (BJA), Hourfar et al. treated their patients with two different fixed functional appliances (the Herbst appliance and the Functional Mandibular Advancer). Moreover, they measured the hyoid position from Sella, Nasion, Gonion, and Menton, but not from the mandibular plane (H-ML), which we suggest is the more suitable measurement in the context of functional orthodontic treatment.

The measurements taken from H to ML more accurately reflect changes in the hyoid position resulting from functional appliances, as these measurements are independent of growth-related confounders and the desired biomechanical influences on the vertical jaw relationship. The use of different measurements thus emphasizes the need to standardize cephalometric reference points for the hyoid positional changes in orthodontics [5, 22, 28–30].

Functional orthodontic treatment typically results in a bite opening, which increases the lower facial height, leading to a caudal displacement of the mandible, and consequently, the hyoid bone is displaced in the same direction. Additionally, growth-related increases in lower facial height, including eruption of the second molars, can further contribute to the caudal displacement of the mandible and the hyoid bone. Hourfar et al. [19] found a caudal displacement of the hyoid after class II treatment with Herbst appliances, measuring + 4.72 ± 7.24 mm from Nasion and + 5.03 ± 7.07 mm from Sella.

Similarly, our results showed a caudal displacement of the hyoid, measuring + 2.14 ± 7.54 mm from Nasion and + 3.06 ± 3.88 mm from Sella, which can be explained by the desired bite opening in patients with a horizontal growth pattern. However, the measurement from the hyoid to the mandibular plane (H-ML) showed a significant reduction in distance after therapy (−1.68 ± 2.89 mm; *P* = 0.003). This approximation of the hyoid to the mandible was also reflected in the significant decrease in the angle measured from the mandibular plane to the hyoid (−2.49 ± 5.50°; *P* = 0.017) (Table 3).

Linear measurements taken from the points aC2, aC3, and aC4 revealed a significant anterior displacement of the hyoid, with the greatest displacement observed at aC4 (Table 3). These results are consistent with those reported in literature [19, 31].

The clinical relevance of these findings extends beyond orthodontics, particularly regarding the pathophysiology of OSA. An inferiorly positioned hyoid bone has been identified as a risk factor for OSA in both pediatric and adult populations [13, 32, 33]. In our study, pretreatment H-ML measurements (12.46 ± 4.89 mm) were within the range associated with increased OSA severity, as reported by Au et al. [34]. Their research showed progressively higher H-ML values across non-OSA, mild OSA, and moderate-to-severe OSA groups: 8.3 mm, 12.1 mm, and 13.1 mm, respectively. Posttreatment, the H-ML values in our cohort approximated those of the non-OSA group, suggesting that functional orthopedic treatment may help normalize hyoid position and potentially reduce OSA risk [30, 35]. These findings support the hypothesis that functional appliances not only produce skeletal changes but may also have preventive effects on upper airway morphology. By improving mandibular position and function, such appliances may reduce the likelihood of upper airway collapse during sleep - a critical factor in the pathogenesis of OSA.

### Strengths and limitations

A key strength of this study is its innovative design, which stratified patients based on their mandibular length response to the same functional appliance. This comparison was based on prior findings that the skeletal mandibular response to BJA therapy differed in relation to specific clinical parameters [20]. This stratification allowed for a targeted analysis of hyoid positional changes in relation to mandibular advancement. As our analysis was exploratory rather than validation-based, we deliberately selected the median change in co-pg as the cut-off point. The median enables the formation of equally sized subgroups, ensuring balanced statistical power and comparability between groups. This approach should be regarded as an exploratory strategy for subgroup stratification.

To further substantiate this rationale, we split the entire cohort according to the median change in co-pg after treatment and calculated the mean change in co-pg within each subgroup (6.7 mm vs. 3.8 mm). Recognizing that part of this change reflects physiological growth, we compared our findings to the data reported by Baccetti et al. [26] for subjects with a similar skeletal maturity. After adjusting our values for the expected growth derived from their control groups, the treatment-related effect in our sample corresponded to Δco-pg increases of approximately 4.2 mm versus 1.8 mm. This is in close agreement (within ± 0.5 mm) with the ranges described by Baccetti et al. [26]. Notably, in their study, the group with a treatment effect of around 4.8 mm comprised patients treated around the pubertal growth peak and exhibited a high skeletal response to therapy. In contrast, the group with a treatment effect of 1.8 mm consisted of patients treated before the pubertal growth spurt and showed only minimal to moderate response. Our classification of subgroups therefore reflects this biologically meaningful distinction.

Another major strength was the reduction of confounding variables. The study cohort was homogeneous in age and skeletal maturation, which addressed a common limitation of earlier studies. This age-matching is particularly relevant, as previous research has shown that hyoid morphology and its clinical implications for OSA vary significantly between early childhood and adolescence [36].

Furthermore, our prospective study design ensured that treatment adherence was objectively monitored using temperature-sensitive microsensors. Wear-time data were collected every six weeks, and patients received regular feedback. To ensure high adherence, individuals with an average daily wear time of less than 10 h were excluded from analysis. This strict inclusion criteria effectively eliminates adherence as a potential confounding variable.

Nevertheless, some limitations must be acknowledged. We only included patients with horizontal or neutral growth patterns, as functional appliances tend to be less effective in individuals with vertical growth and clockwise mandibular rotation. This selection was based on prior clinical and scientific evidence showing more favourable outcomes in counterclockwise mandibular rotation or neutral mandibular growth pattern [37, 38].

Even within this selected cohort, substantial interindividual variation in skeletal response was observed. Notably, patients with a moderate mandibular response often presented with a more caudally positioned hyoid bone at baseline and exhibited a tendency toward vertical growth.

This raises the hypothesis that an initially lower hyoid position may restrict mandibular advancement due to impaired neuromuscular adaptation. Such a position is often associated with oral dysfunctions, such as low tongue posture, and may indicate an imbalance between the suprahyoid and infrahyoid muscle groups. Hyperactivity of the infrahyoid muscles, combined with insufficient tone in the suprahyoid and genioglossus muscles, could stabilise the hyoid bone in a caudal position. This unfavourable muscular pattern may hinder forward translation of the mandible during functional treatment by counteracting the anterior repositioning forces generated by functional appliances. Furthermore, an inferior hyoid position could reduce the efficiency with which the tongue contributes to mandibular advancement, thereby further limiting the skeletal response to functional therapy. Future studies should investigate the supra- and infrahyoid muscles, as well as the relationship between orofacial dysfunctions and an inferior hyoid position during functional orthopaedic treatment.

Although the positional changes of the hyoid bone observed in this study may imply a reduction in upper airway collapsibility, no formal screening or diagnosis of OSA were performed at baseline or posttreatment. Thus, any interpretation regarding OSA risk must be regarded as anatomical and speculative, and confirmation in future studies including poly(somno)graphic assessments is required.

Despite these limitations, the findings of this study contribute to a better understanding of how functional appliance therapy affects hyoid bone position in a growth- and function-dependent manner [39]. They also support the view that orthodontic interventions may play a preventive role in upper airway health, particularly in the context of OSA.

## Conclusion

Mandibular advancement in patients with skeletal class II resulted in positional changes of the hyoid bone, specifically a cranial and anterior shift. The hyoid bone followed the forward movement of the mandible, with the magnitude of change influenced by the initial hyoid position.

These findings highlight the close anatomical and functional relationship between the mandible and the hyoid bone and provide a foundation for further research into the neuromuscular mechanisms underlying these changes. Given that the hyoid bone may serve as a cofactor in the development and persistence of OSA, the therapeutic implications of this relationship warrant further investigation.

## Data Availability

No datasets were generated or analysed during the current study.

## Abbreviations

OSA: Obstructive sleep apnea
BJA: Sander Bite jumping Appliance
MAD: Mandibular advancement device
Cw: Clockwise
Ccw: Counterclockwise
